# Modelling of inactivation kinetics of *Escherichia coli* and *Listeria monocytogenes* on grass carp treated by combining ultrasound with plasma functionalized buffer

**DOI:** 10.1016/j.ultsonch.2022.106086

**Published:** 2022-06-28

**Authors:** Okon Johnson Esua, Da-Wen Sun, Clement Kehinde Ajani, Jun-Hu Cheng, Kevin M. Keener

**Affiliations:** a School of Food Science and Engineering, South China University of Technology, Guangzhou 510641, China; b Academy of Contemporary Food Engineering, South China University of Technology, Guangzhou Higher Education Mega Center, Guangzhou 510006, China; c Engineering and Technological Research Centre of Guangdong Province on Intelligent Sensing and Process Control of Cold Chain Foods, & Guangdong Province Engineering Laboratory for Intelligent Cold Chain Logistics Equipment for Agricultural Products, Guangzhou Higher Education Mega Centre, Guangzhou 510006, China; d Food Refrigeration and Computerized Food Technology (FRCFT), Agriculture and Food Science Centre, University College Dublin, National University of Ireland, Belfield, Dublin 4, Ireland; e School of Engineering, University of Guelph, Canada

**Keywords:** Mathematical model, Plasma species, Akaike information criterion, Accuracy factor, Bias factor

## Abstract

Linear (first-order) and non-linear (Weibull, biphasic, and log-logistic) models were evaluated for predicting the inactivation kinetics of *Escherichia coli* and *Listeria monocytogenes* on grass carp treated by a novel technique (UPFB) combining ultrasound (US) with plasma functionalized buffer (PFB). Results showed that UPFB was more effective for inactivating bacteria when compared with individual applications of US or PFB with reductions of 3.92 and 3.70 log CFU/g for *Escherichia coli* and *Listeria monocytogenes*, respectively. Compared with the linear model, the three non-linear models presented comparable performances and were more suitable for describing the inactivation kinetics with superior adj-R^2^ (0.962–0.999), accuracies (0.970–1.006) and bias factors (0.995–1.031), and by assessing the strengths of evidence, weights of evidence and evidence ratios for the models, the biphasic model was identified as the best fit model. The current study provided new insights into the effective evaluation of decontamination methods.

## Highlights

• DBD plasma system was a significant source of reactive chemistries.
• US and PFB caused 0.26–2.88 log CFU/g reductions of bacterial population in fish.
• UPFB presented synergy with improved reductions of 1.98–3.92 log CFU/g.
• Non-linear models were more accurate for describing bacterial inactivation.
• Biphasic model was the best fit for predicting bacterial inactivation kinetics.

## 1 Introduction

Pathogenic *Escherichia coli* (*E. coli*) and *Listeria monocytogenes* (*L. monocytogenes*) are of global concern owing to their implications in severe illness outbreaks with significant health threats and economic losses [1]. *E. coli* is an enteric human bacterial pathogen responsible for differential gastrointestinal diseases like thrombotic thrombocytopenic purpura and haemorrhagic colitis, while *L. monocytogenes* is a ubiquitous non-spore-forming bacteria that can grow at low temperatures and is responsible for listeriosis with mortality rates of up to 30% [1], [2], [3]. Popular aquaculture species like grass carp, which is the fish species with the largest reported production in aquaculture globally with over five million tonnes per year, can acquire these pathogens from polluted waters and unhygienic handling and processing [4]. Therefore, effective sanitizing approaches are necessary for guaranteeing safe and quality supply and minimizing health hazards.

Conventional washing treatments with chemical sanitisers like peroxyacetic acid and chlorine are important steps during fish processing and have been the standard practice for eliminating pathogens [5], [6]. However, the generation of carcinogenic and toxic disinfection byproducts like trihalomethanes, haloacetic acids, haloacetonitriles, and haloketones, regulatory issues and undesirable changes in product quality have limited their applications [7].

Cold plasma based sanitizers such as plasma functionalized water (PFW), plasma functionalized mist (PFM), plasma functionalized lactic acid (PFLa), and plasma functionalized buffer (PFB) have emerged as eco-friendly washing alternatives to traditional chemical sanitisers [8], [9], [10], [11], [12], [13]. These eco-friendly sanitisers are obtained by exposing water, water mist, and solutions of organic acids and buffers to plasma discharge, and the reactive oxygen and nitrogen species (RONS) generated in these solutions have been reported to exhibit antimicrobial effects on food pathogens. In addition, their strong oxidative potential from reduced pH, etching and ionic bombardment of charged electrons and ultraviolet photons can damage cell wall membrane and degrade RNA and DNA of microbial cells, leading to cell lysis [10], [14], [15], [16], [17]. On the other hand, ultrasound (US) can induce bubble collapse and pyrolysis reactions, and elevate shear actions and micro-cracks in bacterial cell membranes [18], [19], [20], [21], [22], [23].

In our earlier studies [6], [24], [25], [26], the capability of a novel technique (UPFB) of using PFB for decontaminating fish with potential synergy when combined with ultrasound for improving efficiency was demonstrated as such combination approach can generate additional RONS and facilitate the actions of PFB. For further improving the process design and the objective assessment of food safety, it is necessary to determine the inactivation kinetic models for the reliable illustration and prediction of the dynamics of microorganisms during treatments [4], [27].

Mathematical models can be developed from the combination of microbial knowledge, inactivation kinetics and mathematical principles to fit specific processing conditions for evaluating the economics involved in these treatments [27], [28]. Several models have been postulated for fitting the inactivation data of microorganisms, but they are case-dependent and influenced by environmental factors, microbial species, and processing parameters [4]. The classical first-order kinetic model, which relates only to time is typically used to represent linear inactivation kinetics, but the survival curves of microorganisms may exhibit non-linear states like sigmoid effects, shoulder, and tailing, due to resistance variability and mixed microbial population [27], [29], [30]. The deviations from linearity can be properly accounted for by popular non-linear models like Weibull, biphasic, log-logistic, and log-linear models [27], [28], [30].

Nevertheless, the suitability of mathematical models for predicting the inactivation kinetics of microorganisms during decontamination studies with PFB is yet to be investigated. Therefore, the study aimed at evaluating the linear first-order kinetics and non-linear Weibull, biphasic, and log-logistic models for elucidating the inactivation kinetics of *E. coli* and *L. monocytogenes* on grass carp treated with UPFB and individual US or PFB. The models were further quantitatively ranked using the Akaike information criterion (AIC) and evidence ratio for selecting the most parsimonious model. It is hoped that the findings should provide new insights towards the understanding of the tolerance resistance of pathogenic microorganisms, thus providing theoretical support for the effective applications of US, PFB, and UPFB for seafood products decontamination. To the best of our knowledge, this is the first study comparing US, PFB and UPFB inactivation kinetics of *E. coli* and *L. monocytogenes* on a food substrate and evaluating model performances using evidence-based ranking criteria.

## 2 Materials and methods

### 2.1 Chemicals and reagents

Luria-Bertani (LB) broth, tryptic soy broth with 0.6% yeast extract (TSB-YE), tryptic soy agar with 0.6% yeast extract (TSA-YE), nutrient agar (NA) and sorbitol McConkey agar (SMA) were provided by Huankai Microbial Science and Technology Co., Ltd. (Guangzhou, China). Sodium chloride (NaCl), sodium hydrogen phosphate (Na_2_HPO_4_), nalidixic acid (C_12_H_12_N_2_O_3_) and citric acid (C_6_H_8_O_7_) were procured from Aladdin Biochemical Technology Co., Ltd. (Shanghai, China). Glycerol (C_3_H_8_O_3_) and ethanol (CH_3_CH_2_OH) were purchased from Tianjin Fuyu Fine Chemical Co., Ltd. (Tianjin, China). Hydrochloric acid (HCl) was obtained from Guangzhou Rongman Technology Co., Ltd. (Guangzhou, China). Sulphanilamide (C_6_H_8_N_2_O_2_S), sulfamic acid (HSO_3_NH_2_) and N-(1-naphthyl)-ethylenediamine dihydrochloride (C_12_H_16_C_l2_N_2_) were provided by Macklin Biochemical Co., Ltd. (Shanghai, China).

### 2.2 Bacteria strains and inoculum preparation

Bacterial strains of *E. coli* ATCC25922 and *L. monocytogenes* ATCC19115 were supplied by Guangzhou Microbial Culture Centre (Guangzhou, China) and maintained at −80 ℃. For preparing the stock culture, the freeze-dried strains were activated overnight in 250 mL LB broth for *E. coli* and 250 mL TSB-YE for *L. monocytogenes* at 37 ℃ with constant agitation in an orbital shaker (WSZ-10A, Shanghai Yiheng Technology Co., Ltd., Shanghai, China) at 150 rpm, and maintained as frozen stocks in LB broth containing 50% C_3_H_8_O_3_ at −80 ℃ in a freezer (DW-86L386, Haier Special Electrical Appliances Co., Ltd., Qingdao, China) pending use. The frozen stocks were cultured twice in NA for *E. coli* and TSA-YE for *L. monocytogenes* and incubated overnight for 18 – 24 h at 37 ℃ for obtaining pure strains. Loopfuls of the pure isolated colonies were subsequently suspended in LB broth for *E. coli* and in TSB-YE for *L. monocytogenes* and incubated at 37 ℃ for 18–24 h with constant agitation at 150 rpm. Subsequently, 10 mL of each bacteria culture was centrifuged (JW-3024HR, Anhui Jiaven Equipment Industry Co., Ltd., Hefei, China) for 10 min at 3380 × *g* and 4 ℃ for harvesting bacteria cells, followed by repeated washing and re-suspension of the pellets in a sterile solution of 0.85% NaCl for three times to obtain the working inoculum. The final cell population of the inoculum was in the range of 10^6^–10^7^ CFU/mL, confirmed by the plate count method using SMA for *E. coli* and TSA-YE for *L. monocytogenes.*

### 2.3 Sample preparation and inoculation

Farmed grass carp (deceased) from comparable breeding environments weighing 1.5–2.4 kg and 15–20 cm in length were obtained from a local supermarket (Guangzhou, China). They were washed in running tap water, eviscerated and filleted to obtain experimental samples of 4.0 x 2.0 x 2.0 cm and 10 ± 0.5 g. Fillets were spread on sterile aluminium foil in a biosafety cabinet (BSC-1100IIB2-X, Jinan Biobase Biotech Co., Ltd., Jinan, China) and separately inoculated by aseptically transferring 100 µL of the prepared inoculum of *E. coli* and *L. monocytogenes* to the surfaces with the aid of a spreader. The inoculated samples were air-dried in the biosafety cabinet for 1 h at room temperature of 25 ℃ for allowing bacterial attachment to present samples with initial bacteria concentration in the range of 6–7 log CFU/g.

### 2.4 Generation and characterization of PFB

Functionalization of the buffer was achieved by exposing 20 mL of citrate–phosphate buffer solution (CBS), prepared by mixing 11.01 g C_6_H_8_O_7_ and 4.29 g Na_2_HPO_4_ in 500 mL of distilled water (DW) to cold plasma discharge. For the current study, a dielectric barrier discharge (DBD) cold plasma reactor (CTP-2000K, Nanjing Suman Electronics Co., Ltd., Nanjing, China) consisting of low and high voltage electrodes, separated by a circular quartz glass dielectric barrier was employed [31], [32], [33]. The system was operated under atmospheric pressure and room temperature of 25 °C with a resonance balancing, frequency, voltage and exposure time of 1.05 A, 10 kHz, 70 V, and 8 min, respectively. A distance of 5 mm was maintained between the liquid surface and plasma discharge, and the optical emission spectroscopy technique described in our previous study [6] was employed for determining the profile of plasma reactive species in the gaseous phase. The temperature of the generated PFB reached 49.75 ℃, and the application of cold plasma is usually recommended at ambient temperatures or <40 ℃ for maximizing the nonthermal effect [13]. Thus, a cooling time of 2 min was allowed to minimize the temperature effects during the treatment of the fish, and the physicochemical properties of the PFB were characterized as described in our previous study [6].

### 2.5 Inactivation of bacteria on grass carp by US, PFB and UPFB

Ultrasound treatment (US) was achieved by immersing inoculated samples in beakers containing 20 mL of DW and placed to a depth of 80 mm from the bottom and equidistant from the walls of a bath-type sonochemical reactor (SB25-12D, Ningbo Xinzhi Ultrasonic Equipment Co., Ltd., Ningbo, China). The reactor tank had internal dimensions of 500 x 300 x 150 mm (L x W x H) and was operated at 40 kHz, 500 W, and room temperature of 25 ℃ for the treatment time of 3, 6, 9, 12, and 15 min, and the treated samples were designated as UDWS3, UDWS6, UDWS9, UDWS12, and UDWS15, respectively. Heat is typically generated during ultrasound treatments in liquid media, and the temperature change of the system was used for evaluating the power dissipated by the reactor and the effective acoustic intensity (E_ai_) as described in our previous study [25]. The final temperatures of the system were 25.50, 26.10, 26.80, 27.50, and 28.40 ℃ for sonication times of 3, 6, 9, 12, and 15 min, with $E_{\mathrm{ai}}$ of 11.63, 12.79, 13.96, 14.54, and 15.82 W/L respectively. For PFB treatements, the inoculated samples were immersed in the PFB contained in beakers and placed on an orbital shaker operating at 150 rpm and room temperature of 25 ℃ for 3, 6, 9, 12, and 15 min, and the treated samples were designated as PFBS3, PFBS6, PFBS9, PFBS12, and PFBS15, respectively. Combination treatments (UPFB) were achieved by immersing the samples in PFB and subjected to US as described above, and the treated samples were designated as UPFBS3, UPFBS6, UPFBS9, UPFBS12, and UPFBS15, respectively. For the control, samples were immersed in DW for the above treatment times and designated as DWS3, DWS6, DWS9, DWS12, and DWS15, respectively.

### 2.6 Enumeration of surviving bacteria cells

The surviving bacteria cells on grass carp were evaluated from the colony forming unit (CFU) count assay method [6]. Samples were added to 90 mL 0.85% sterile NaCl solution in sterile stomacher bags (Huankai Microbial Science and Technology Co., Ltd., Shanghai, China) and homogenized for 60 s in a stomacher (QIQIAN-08, Qiqian Electronic Technology Co., Ltd., Shanghai, China). Thereafter, 10-fold serial dilutions were prepared from the homogenized solutions using sterile 0.85% NaCl solution, and 100 µL of serially diluted solutions were spread on SMA plate for *E. coli* and on TSA-YE (supplemented with 200 µg/mL nalidixic acid) plate for *L. monocytogenes*. These plates were incubated at 37 ℃ for 24 h and the surviving bacteria colonies were enumerated and expressed as log CFU/g.

### 2.7 Evaluation of synergy during treatment

The combined treatment (UPFB) was evaluated for possible synergy in improving the inactivation efficiency by comparing with the corresponding individual inactivation. Synergistic, antagonistic or no effects were established by positive, negative or zero values using the equation below:

(1) $$\mathrm{Effect}=Y_{\mathrm{ij}}-\left(Y_{i}+Y_{j}\right)$$

where Y is microbial log reduction, i and j are US and PFB treatments, respectively.

### 2.8 Models for bacteria inactivation

The first-order kinetic model is widely accepted for predicting the inactivation patterns of microorganisms, and the model assumes a similar resistance level for the microbial population with a linear relationship existing between the number of surviving cells and treatment time [30], [34]. The first-order kinetic model is typically described by the following equation:

(2) $$\log\frac{N_{t}}{N_{o}}=-\frac{t}{D_{T}}$$

where $N_{o}$ and $N_{t}$ (CFU/g) are the initial and surviving populations of bacteria after treatment time t (min), respectively. $D_{T}$ (min) is the decimal reduction time, which indicates the time required to inactivate 90% of the bacterial population.

The first-order kinetic model is usually valid for only log-linear inactivation patterns, but microbial inactivation curves tend to exhibit non-log-linear relationships [3], thus, the Weibull, biphasic and log-logistic models are normally employed for analyzing non-log-linear survival curves. The Weibull model assumes that bacteria consist of subpopulations with different levels of sensitivity described by a specific time and non-linear upward and downward concavities. For the Weibull model, the corresponding number of surviving bacteria after treatment is described by the semi-logarithm equation with temperature-dependent coefficients as shown below [30], [35], [36]:

(3) $$\log\frac{N_{t}}{N_{o}}=-bt^{n}$$

where b is the characteristic slope or scale parameter (min^−1^) and n is the dimensionless shape parameter.

The Weibull inactivation curve is assumed to possess upward and downward concavity when the shape parameter is <1 and >1, suggesting a logarithmic curve with a “tailing or increasing resistance” and “shoulder or decreasing resistance” portion, respectively, attributed to variations in the resistance of bacteria to treatment.

The biphasic model is employed for predicting broken curves and assumes the coexistence of two population groups with different resistance levels and different inactivation phases. It comprises three temperature-dependent parameters namely the first and second inactivation rates and the phase transfer time, and is expressed in the following form [4], [27], [28], [36]:

(4) $$\log N_{t}=\log N_{o}+\log\left(k*e^{-\alpha t}+\left(1-k\right)*e^{-\beta t}\right)$$

where k and 1-k are the fractions of treatment-resistant and treatment-sensitive cells, respectively, and α (min^−1^) and β (min^−1^) are their corresponding inactivation rate constants.

On the other hand, the log-logistic model is a vitalistic model for predicting non-linear inactivation kinetics and can be used for comparing the Weibull model, and the model parameters are given below [30], [37], [38]:

(5) $$\log\frac{N_{t}}{N_{o}}=\frac{A}{1+e^{4\sigma (\tau -logt)/A}}+\frac{A}{1+e^{4\sigma (\tau +6)/A}}$$

where A is the difference between the upper and lower asymptotes (log CFU/g), σ is the maximum inactivation rate (log (CFU/g)/log min), and τ is the log time to achieve the maximum inactivation rate (log min).

### 2.9 Model assessment and validation

The performances of the above models were evaluated based on the sum of squared errors (SSE), root mean square error (RMSE), coefficient of determination (R^2^) and adjusted coefficient of determination (adj-R^2^) as expressed below [4], [39].

(6) $$\mathrm{SSE}=\sum_{0}^{j-1}{\left(predicted\;value-observed\;value\right)}^{2}$$

(7) $$\mathrm{RMSE}=\sqrt{\frac{\sum {\left(predicted\;value-observed\;value\right)}^{2}}{j-m}}$$

(8) $$R^{2}=\frac{\sum {\left(predicted\;value-average\right)}^{2}}{\sum {\left(observed\;value-average\right)}^{2}}$$

where j is the number of observations in the curve, m is the number of parameters in a model.The models were validated from the accuracy (A_f_) and bias (B_f_) factors as expressed below [27], [40]:

(9) $$A_{f}=10^{\frac{\sum \left|log(predicted\;value/observed\;value)\right|}{j}}$$

(10) $$B_{f}=10^{\frac{\sum log\left(observed\;value/predicted\;value\right)}{j}}$$

The A_f_ shows the closeness of predicted values to the observed values with values close to 1 indicating slight deviations, while B_f_ assesses the degree of prediction with values >1 and <1 indicating over prediction and under prediction from the observed values, respectively [27].

Since several models may fit a set of experimental data with a virtually equivalent level of efficiency, the Akaike information criterion (AIC) is employed for identifying the most appropriate model [40]. The AIC computes the goodness of fit (accuracy) and variability (precision) of the models for quantitative ranking, thereby selecting the most parsimonious model [41]. The AIC is calculated as follows:

(11) AIC=jlnSSE-jlnj+2m

The smaller the AIC, the better the ability of the corresponding model for describing the data set. The method for computing AIC also allows the ranking of the models, known as the quick “strength of evidence” comparison [27], [40]. By computing the relative AIC differences between a candidate model (AIC_i_) and the Akaike best-ranked model (model with the lowest AIC value), the Akaike increment (Δ_i_) can be used for determining the relative support for each candidate model and is computed as shown below [27], [41]:

(12) $$\Delta_{i}={\mathrm{AIC}}_{i}-{\mathrm{AIC}}_{\min}$$

where ${\mathrm{AIC}}_{\min}$ is the Akaike best-ranked model or model with the lowest AIC value.

The model with Δ_i_ ≡ Δ_min_ ≡ 0 is judged to be the best, and the relative positions of models in a data set are evaluated thus: $\Delta_{i}\leq 2$ shows substantial support and the models should be given consideration when making inferences, $4\leq \Delta_{i}\leq 7$ shows relatively less support, and $\Delta_{i}\geq 10$ shows essentially no support and may not be given consideration. Furthermore, Δ_i_ can be used to construct the weight of evidence or Akaike weights (W_i_), which is interpreted as the probability of the model being the best-ranked among the sets of candidate models, and is calculated as shown below [27], [41]:

(13) $$W_{i}=\frac{exp\left(-\frac{\Delta_{i}}{2}\right)}{\sum_{i=1}^{R}exp\left(-\frac{\Delta_{i}}{2}\right)}$$

where i = 1, 2, …, R, which shows the likelihood of the model and R represents the set of candidate models.

The relative likelihood of model *i* versus model *j* is termed as the evidence ratio (E_R_) and by this ratio, a model can be assumed to fit better relative to the other models. E_R_ is evaluated as shown below [41]:

(14) $$\mathrm{Evidence}\;\mathrm{ratio}\left(E_{R}\right)=\frac{W_{1}}{W_{2}}$$

### 2.10 Statistical analysis

OriginPro 9.8.0.200 (OriginLab Corp., Massachusetts, USA) was employed for data variance, linear and non-linear analysis, and graphical representation. Data were analyzed in triplicates using analysis of variance (ANOVA) and expressed as the average data ± standard error of measurement (SEM). The significant differences between means were further separated by the post-hoc Turkey test at a significance level of *p* < 0.05 for all pairwise multiple comparisons.

## 3 Results and discussion

### 3.1 Plasma reactive species and characterization of PFB

**Fig. 1:**
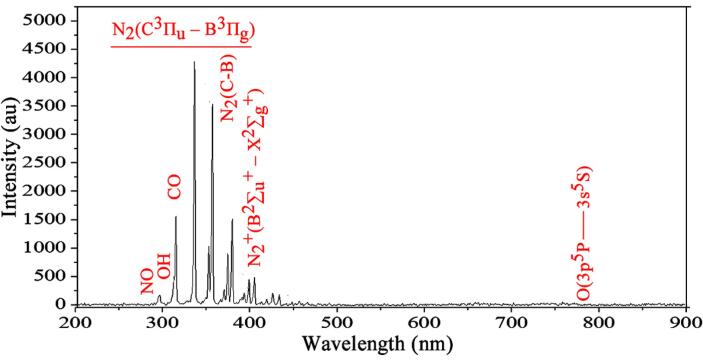
Optical emission profile of the DBD plasma system.

The optical emission profile of the DBD reactor is presented in Fig. 1. Five distinct peaks appeared within the wavelength range of 338–405 nm, which indicated the presence of reactive nitrogen species of the second positive system of N_2_(C-B) and the first negative system of N_2_^+^ (B-X) [42]. In addition, NO emission, reactive oxygen species such as OH, CO, and O atoms linked to the quenching O(^3^P) and O(^5^P) were observed at 297, 300, 313, and 780 nm, respectively. The reactive species reported in the current study were similar to reports in previous studies [3], [9], [34], [43], and indicated that the DBD plasma system was a significant source of reactive chemistries [44], [45], [46]. Further interactions between the reactive species and active ions in the solution at the gas–liquid interface can generate other RONS and acidic species like HNO_3_, HNO_2_, and ONOOH in PFB, which have been reported to show potent antimicrobial effects against several microorganisms [47], [48].

The concentrations of O_3_ and H_2_O_2_ in PFB were changed to 0.38 mg/L and 2.26 µM from 0.68 mg/L and 0.006 µM for CBS, respectively, while the concentration of NO_2_^–^ and NO_3_^–^ were 3.38 and 11.94 mM, respectively. O_3_ and H_2_O_2_ show excellent bactericidal properties that may result in the oxidation of membrane lipids, proteins and DNA in bacteria cells. NO_2_^–^ and NO_3_^–^ have been reported to exhibit low antimicrobial properties, but react in the presence of H_2_O_2_ to generate OH radicals, NO•, NO_2_ and acidic species as shown above, which were responsible for acidifying CBS from a pH of 3.75 to 2.59 in PFB [34]. Studies have suggested that OH, ONOOH and the ionic ONOO^–^ exhibit potent hydroxylation, oxidation, and nitration potentials against the biomolecules of bacteria cells for inhibiting growth [6], [34], [49]. The oxidation–reduction potential (ORP) and electrical conductivity (EC) of PFB were also determined and the values increased to 630.79 mV and 8.72 mS/cm after plasma exposure from CBS values of 414.25 mV and 5.61 mS/cm, respectively. The increases in ORP and EC were attributed to the generation of numerous RONS and reflected the nature of ions generated, concentration of oxidizers, and degree of electron activity in PFB, which play significant etching roles in destroying microbial defence and membrane structure [25], [48], [50], [51]. The results of the current study were similar to reports for PFW [30], [48], PFLa [10], and PAM [9].

### 3.2 Microbial inactivation under individual and combination treatments

**Fig. 2:**
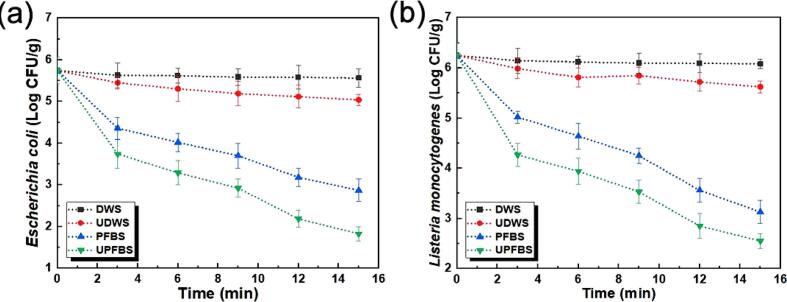
Surviving populations of (a) *Escherichia coli* and (b) *Listeria monocytogenes* with time during treatments. Values represent the mean ± standard error of measurement (n = 3).

The surviving population of *E. coli* and *L. monocytogenes* on grass carp after treatments is presented in Fig. 2. Inoculated grass carp presented initial microbial counts of 5.75 for *E. coli* and 6.25 log CFU/g for *L. monocytogenes*. The counts of both pathogens were significantly reduced on grass carp and the inactivation efficiency was dependent upon the immersion and treatment time. The highest level of inactivation was observed after inactivation for 15 min at values of 0.19, 0.71, 2.88, and 3.92 log CFU/g for *E. coli*, and 0.17, 0.63, 3.12, and 3.70 log CFU/g for *L. monocytogenes*, respectively for DWS, UDWS, PFBS, and UPFBS. It was evident that the bactericidal effects of US alone were limited, but comparable with the inactivation of *Staphylococcus* app., *L. monocytogenes*, and natural microbiota on salmon and chicken, and the disparity in results could be attributed to equipment type, variations in tested parameters and resistance of bacteria [52], [53]. The mechanism of bacteria inactivation by US was either through mechanical disruption of cell walls by cavitation-induced shockwaves, or lethal injury from changes in permeability as a result of increased cellular volume during pore formation [54]. Bath-type sonochemical reactors have been reported to show uneven distribution and spatial variations in cavitation intensity [55], and this might be responsible for the low efficiency observed for US.

Nevertheless, the results confirmed the antibacterial properties of PFB and suggested that US could further promote the reduction of bacteria when combined with PFB. The log reductions from the combination treatments (UPFB) were higher when compared with the sum of the log reductions from individual treatments (US + PFB) for both pathogens, with the exception of *L. monocytogenes* during 15 min treatment. Thus, the interactions between treatments presented potential synergistic effects in the range of 0.18–0.50 log CFU/g for both pathogens, comparable with studies combining US and sanitisers [52], [53]. The antimicrobial mechanism of plasma functionalized liquids is yet to be fully elucidated, but bacterial resistance is weakened in acidified environments, and CBS was acidified to a pH of 2.59 during the generation of PFB. The weakened bacterial resistance might improve the penetration of RONS in PFB for compromising membrane cellular integrity of bacteria cells to allow for chemical and physical sterilization [6], [34]. It has been demonstrated that RONS can damage the C-N, C–C, and C-O bonds of lipopolysaccharide and peptidoglycan in bacterial cell walls to the molecular structure, and the oxidative stress to the double bonds of ROS-saturated fatty acids in cell membranes can prevent the movement of biomolecules to cause cell lysis [34]. Furthermore, US might cause membrane disruption during the combination treatments from pyrolysis reactions that produced additional free radicals to increase the ORP of PFB. Coupled with the possible rupture of the cytoplasmic membrane from cavitation-induced bubble implosion, bacterial cells might become more susceptible to RONS from PFB, hence the inactivation during the combination treatments was improved [53]. Similar trends and results were demonstrated for various pathogens on chicken during decontamination with US and PFW [19], PFLa [10], and thawing with US and PFW [47].

### 3.3 Evaluation and validation of model performance

**Fig. 3:**
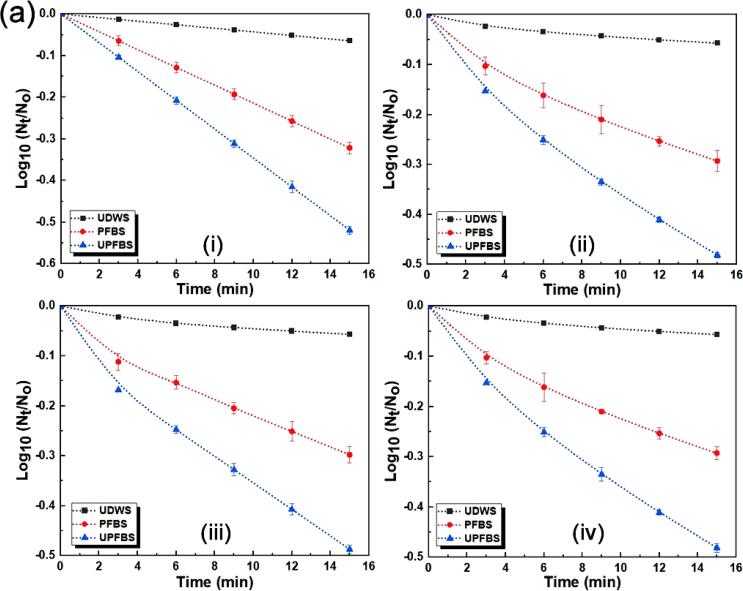

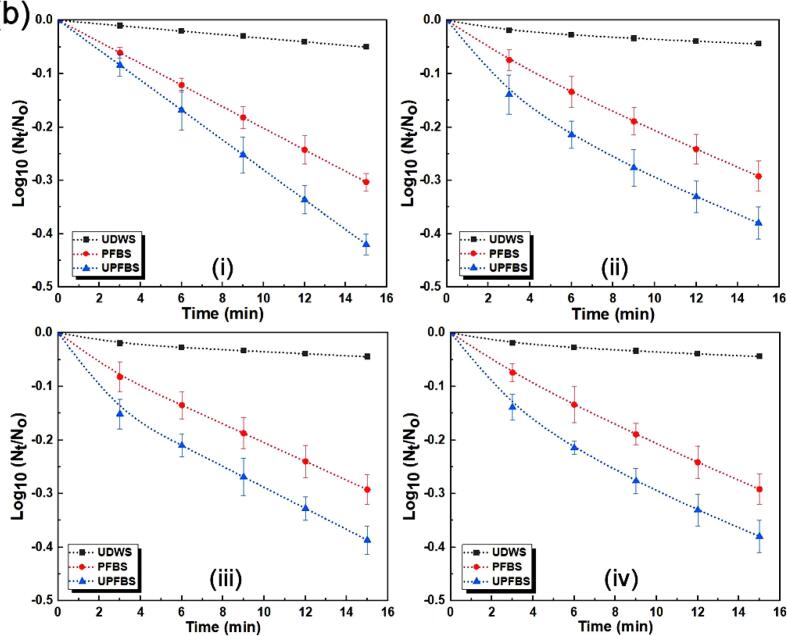
Survival curves of inactivation kinetics data for (a) *Escherichia coli* and (b) *Listeria monocytogenes* fitted with different models: (i) first-order kinetics (ii) Weibull (iii) biphasic and (iii) log-logistic models.

Predictive modelling of the inactivation kinetics of microorganisms during decontamination provides useful information for the quantitative assessment of microbial risk, in addition to offering tools for comparing the significance of different processing technologies on microbial population reduction [3], [34]. Fig. 3 presents the kinetic profiles of the survival curves of *E. coli* and *L. monocytogenes* fitted to the different models, and the statistical indices utilized for assessing the performance of the models are summarized in Table 1. The studied models presented comparable fittings to the inactivation data as indicated by the error values, accuracy indices, and coefficients of determination, with the exception of the first-order kinetic model for UDWS. The SSE, RMSE, A_f_ and B_f_ values were within the typical range for microbial decontamination studies [3], [27], [30]. Generally, R^2^ and adj-R^2^ values close to 1 and smaller SSE and RMSE values indicate the better fitting ability of a particular model [28], [38].

**Table 1:** Parameters and validation indices for evaluating model performance.

Models	Parameters and indices	UDWS	PFBS	UPFBS	UDWS	PFBS	UPFBS
		*Escherichia coli*			*Listeria monocytogenes*		
First-order kinetics	R^2^	0.879	0.926	0.943	0.829	0.974	0.910
	adj-R^2^	0.879	0.926	0.943	0.829	0.974	0.910
	SSE	0.0091	0.228	0.593	0.0056	0.202	0.388
	RMSE	0.0073	0.0288	0.0424	0.0066	0.0174	0.0411
	A_f_	0.849	0.862	0.875	0.837	0.914	0.850
	B_f_	1.179	1.160	1.143	1.195	1.094	1.176

Weibull	R^2^	0.999	0.986	0.980	0.962	0.983	0.980
	adj-R^2^	0.998	0.983	0.975	0.953	0.978	0.975
	SSE	0.0094	0.231	0.599	0.0057	0.203	0.395
	RMSE	0.0008	0.0139	0.0287	0.0032	0.0159	0.0220
	A_f_	1.006	0.986	0.979	1.002	0.970	0.982
	B_f_	0.995	1.014	1.021	0.998	1.031	1.018

Biphasic	R^2^	0.999	0.995	0.989	0.967	0.988	0.990
	adj-R^2^	0.998	0.991	0.981	0.945	0.980	0.984
	SSE	0.0094	0.231	0.600	0.0057	0.203	0.396
	RMSE	0.0006	0.0100	0.0247	0.0035	0.0150	0.0176
	A_f_	1.003	0.996	0.992	1.005	0.989	0.991
	B_f_	0.997	1.004	1.008	0.995	1.011	1.009

Log-logistic	R^2^	0.999	0.986	0.979	0.962	0.982	0.980
	adj-R^2^	0.999	0.977	0.966	0.937	0.970	0.967
	SSE	0.0093	0.230	0.599	0.0057	0.201	0.395
	RMSE	0.0006	0.0162	0.0333	0.0037	0.0185	0.0256
	A_f_	1.004	0.986	0.979	1.002	0.970	0.982
	B_f_	0.996	1.014	1.021	0.998	1.031	1.018

UDWS: ultrasound treatment, PFBS: immersed in plasma functionalized buffer, UPFBS: immersed in plasma functionalized buffer and treated with ultrasound, SSE: sum of squared error, RMSE: root mean square error, A_f_: accuracy factor, B_f_: bias factor. Values are reported at *p* < 0.05.

The data for the first-order kinetic model suggested that the model did not satisfactorily explain the inactivation patterns of *E. coli* and *L. monocytogenes* for UDWS, with adj-R^2^ of 0.879 and 0.829, respectively, similar to the report of Kim et al. [3]. However, the model presented a fairly good fit and explanation of the reduction patterns for PFBS and UPFBS, with adj-R^2^ ≥ 0.910, SSE ≤ 0.593, and RMSE ≤ 0.0423. The Weibull model adequately described the inactivation data for both pathogens as it accounted for >96.2% of the response variability, i.e., adj-R^2^ ≥ 0.962 with SSE ≤ 0.599 and RMSE ≤ 0.0287 for all the treatment methods. The biphasic model also performed well for both pathogens irrespective of the treatment methods employed, and presented adj-R^2^ ≥ 0.980, SSE ≤ 0.600 and RMSE ≤ 0.0247, with the exception of *L. monocytogenes* for UDWS, which displayed adj-R^2^ of 0.945 and very low SSE and RMSE of 0.0057 and 0.0035, respectively. Likewise, the inactivation of *L. monocytogenes* for UDWS presented a relatively low adj-R^2^ of 0.937 for the log-logistic model, while the other treatments displayed adj-R^2^, SSE, and RMSE in the range of 0.966 – 0.999, 0.0093 – 0.599, and 0.0006 – 0.0333, respectively, depending on the studied pathogen.

Validation indices for the different models showed that the first-order kinetic model presented A_f_ values of 0.837–0.914 and B_f_ values of 1.094–1.195 for all treatments for both pathogens, indicating large deviations from and over prediction of the experimental data, respectively (Table 1). This was in agreement with the relative poor-fitting ability from the fairly low adj-R^2^ observed earlier. The other models exhibited acceptable accuracy with A_f_ and B_f_ close to 1 for some treatments and pathogens, and slightly >1 for the others. Precise agreement between predicted and observed data leads to a bias factor of 1 that signifies model reliability [27]. In particular, the biphasic model provided very good fits for all inactivation curves, presenting A_f_ values of 0.992–1.003 and 0.989–1.005, and B_f_ values of 0.997–1.008 and 0.995–1.011, respectively for *E. coli* and *L. monocytogenes*. The Weibull and Log-logistic models were also reasonably good fits in terms of the B_f_ values, with the exception of PFBS and UPFBS, which showed values slightly higher than 1 at 1.014 and 1.021 for *E. coli* and 1.031 and 1.018 for *L. monocytogenes*, respectively. Overall, the non-linear models presented comparable performances and were better fits for predicting the inactivation kinetics of both pathogens in comparison with the first-order kinetic model.

### 3.4 Kinetic parameters of the different models

#### 3.4.1 First-order kinetic model

The fitting of the experimental data to the different models enabled the estimation of their kinetic parameters and regression coefficients, and the data are presented in Table 2. According to the first-order kinetic model, the D_T_-value provides a quantitative measure of the resistance of microorganisms to the applied lethal agent and represents the time required for 90% or one log-cycle reduction [3], [34]. As observed from the table, the D_T_-values were 233.04, 46.64, and 28.88 min for *E. coli* and 298.54, 49.46, and 35.70 min for *L. monocytogenes*, respectively for UDWS, PFBS, and UPFBS. The results suggested that pathogens were more susceptible to UPFB treatments since their counts were reduced considerably after shorter treatment times, which was consistent with the improved inactivation observed in the experimental data. Bacteria spores can show great resistance to singular treatments but may be readily inactivated at improved rates with combination or hurdle technologies [1], [38].

**Table 2:** Estimated kinetic and selection of best fit parameters for the different models.

Models	Parameters	UDWS	PFBS	UPFBS	UDWS	PFBS	UFPBS
		*Escherichia coli*			*Listeria monocytogenes*		
First-order kinetics	D_T_ (min)	233.04 ± 17.71	46.64 ± 2.84	28.88 ± 1.58	298.54 ± 27.13	49.46 ± 1.90	35.70 ± 2.40
	AIC	−39.95	−17.62	−11.89	−39.86	−18.35	−14.43
	Δ_i_	0.00	0.00	0.00	0.00	0.00	0.00
	W_i_	0.0499	0.0524	0.0524	0.0525	0.0539	0.0512

Weibull	b (min^−1^)	0.013 ± 0.004	0.050 ± 0.009	0.070 ± 0.016	0.011 ± 0.003	0.029 ± 0.007	0.070 ± 0.014
	n	0.556 ± 0.019	0.651 ± 0.071	0.712 ± 0.092	0.523 ± 0.106	0.850 ± 0.098	0.625 ± 0.083
	AIC	−34.75	−15.54	−9.83	−37.75	−16.32	−12.32
	Δ_i_	2.19	2.08	2.06	2.11	2.03	2.11
	W_i_	0.1496	0.1480	0.1469	0.1505	0.1488	0.1469

Biphasic	k	0.942 ± 0.077	0.834 ± 0.034	0.816 ± 0.047	0.963 ± 0.020	0.860 ± 0.021	0.808 ± 0.034
	α (min^−1^)	0.005 ± 0.001	0.036 ± 0.002	0.061 ± 0.006	0.004 ± 0.002	0.040 ± 0.004	0.045 ± 0.004
	β (min^−1^)	0.63 ± 0.171	10.64 ± 2.33	71.43 ± 8.92	0.34 ± 0.063	6.19 ± 0.001	21.86 ± 0.002
	AIC	–32.75	−13.54	−7.82	−35.75	−14.32	−10.31
	Δ_i_	4.19	4.08	4.07	4.11	4.03	4.12
	W_i_	0.4067	0.4020	0.4013	0.4091	0.4045	0.4020

Log-logistic	A (log CFU/g)	−0.19 ± 0.11	−19.32 ± 6.38	–33.34 ± 9.48	−3.85 ± 0.09	−17.48 ± 4.56	−25.10 ± 8.83
	σ (log (CFU/g)/log min)	−0.08 ± 0.03	−7.30 ± 2.25	−13.79 ± 5.85	−1.17 ± 0.01	−8.64 ± 1.67	−9.11 ± 2.34
	τ (log min)	4.63 ± 0.58	3.94 ± 0.75	3.24 ± 0.53	4.85 ± 1.01	4.05 ± 1.22	3.73 ± 0.59
	AIC	–32.82	−13.57	−7.83	−35.86	−14.37	−10.32
	Δ_i_	4.13	4.05	4.06	4.00	3.97	4.11
	W_i_	0.3938	0.3972	0.3993	0.3879	0.3927	0.3994

UDWS: ultrasound treatment, PFBS: immersed in plasma functionalized buffer, UPFBS: immersed in plasma functionalized buffer and treated with ultrasound, AIC: Akaike information criterion, Δ_i_: Akaike increments, W_i_: Akaike weights. Kinetic parameters are reported as mean ± SEM at *p* < 0.05.

The data also demonstrated that *E. coli* was more susceptible to the treatments, suggesting that the dynamics of bacterial inactivation varied with species. This might be related to the differences in structures, chemical compositions, and molecular organisations of cell walls between gram-positive and gram-negative bacteria, and similar variability in the sensitivity of bacteria species was reported during treatments [27], [54], [56]. Generally, the apparent properties of thick peptidoglycan structure on the cell wall of gram-positive bacteria like *L. monocytogenes* in comparison with gram-negative *E. coli* can offer more resistance to chemical changes for inhibiting inactivation [34], which was evident in the higher D_T_-values for *L. monocytogenes*. The relatively high D_T_-values in the current study, especially for UDWS might be related to the poor fitting ability of the first-order model to the experimental data observed earlier. However, D_T_-values of 33.00 and 32.36 min were reported for *Staphylococcus aureus* (*S. aureus*) and *Bacillus cereus* (*B. cereus*) inactivation, respectively, during DBD plasma treatment of dried blackmouth angler [34]. Values of 15.06–35.01 min were also reported during the inactivation of *B. cereus* by PFW, and the values were dependent on the temperature, the concentration of interfering organic substances, initial spore concentration, and functionalized volume of PFW [30].

#### 3.4.2 Weibull model

The Weibull model is regarded as a simplistic and robust alternative to the linear kinetic model and presents the approach of treating microbial inactivation as the distribution of surviving population associated with variations in treatment intensity or heterogeneous resistance of microorganisms [28], [35]. As shown in Table 2, the Weibull model displayed a distinctive curve with the shape parameter at upward concavity (*n* < 1) for all treatments, at values of 0.556, 0.651, and 0.712 for *E. coli* and 0.523, 0.850, and 0.625 for *L. monocytogenes*, respectively for UDWS, PFBS, and UPFBS. The results were similar to the reports for *Neosartorya fischeri* (*N. fischeri*) inactivation during US and high-pressure processing [37], *E. coli* and *L. monocytogenes* during the combination of US and sanitisers [1], but contrasted the results for *E. coli*, *B. cereus* and natural microbiota during US and thermoultrasound treatments, where two distinctive curves with upward and downward concavities were reported [28], [38]. Concavity can be employed for interpreting inactivation resistance, as upward concavity indicates tailing or increasing resistance and downward concavity suggests shoulder or decreasing resistance [35].

The occurrence of tailing can be attributed to the adaptive behaviour, acquired resistance or self-defence mechanisms adopted by the pathogens for inhibiting cavitation-induced actions of US and oxidative stress from RONS [27]. This is probably associated with the protection from organic compounds in the fish, dead bacteria cells, and the depletion of RONS in PFB with prolonged treatment, and elucidated the increasingly insensitive behaviour of bacteria with less probability of lysis as the treatment time increased, indicative of incomplete inactivation irrespective of the treatment method [27], [38]. However, the pathogens were more resistant to individual treatments (US and PFB) as the combination treatments (UPFB) presented higher *n* values, which was also confirmed from the experimental inactivation data. The reduced resistance with combination treatments was linked to the accumulated damage of bacteria cells from the rigorous cavitation effects of US, which increased the vulnerability of cells to the actions of RONS from PFB described earlier. The *n* values were also higher for *E. coli* with the exception of PFBS for *L. monocytogenes*, further confirming that gram-negative *E. coli* presented less resistance and was more susceptible to treatments, similar to the report of Frohling et al. [57] during cold plasma treatment of a polysaccharide gel. The exception might be related to the comparable inactivation for both pathogens after 9 min, and improved inactivation for *L. monocytogenes* after 12 min during PFB treatment.

Moreover, the *b* value is a scale or slope parameter that relates to the time required for the first decimal reduction of bacteria colony, and higher inactivation rates are associated with increasing slopes [35], [57]. UPFBS presented higher values for both pathogens at 0.070, further confirming the improved efficiency of the combination treatments. Increasing *b* values were also reported for the improved inactivation of *B. cereus* with increasing temperature during PFW [30] and *N. fischeri* during high pressure treatment [37].

#### 3.4.3 Biphasic model

The biphasic model is a derivative of the first-order kinetic model and is often applied for inactivation modelling owing to its consideration of a non-zero activity during prolonged treatments [28]. The model describes broken curves with different subpopulation resistances by taking into consideration a fraction of the survivors that seem to be more resistant (*k*) when compared with the other subset (1*-k*) [27], [28], [58]. Among the treatment methods, US and UPFB presented the highest and lowest *k* values for both pathogens, respectively, underlying the existence of a substantial proportion of resistant cells during US, which could be related to the limited efficiency of US (Table 2). *L. monocytogenes* presented higher fractions of resistant cells (*k*) at values of 0.808 – 0.963 with the exception of UPFBS, and lower inactivation rates for the resistant cells (α) at values of 0.004 – 0.045, indicating the higher resistance of *L. monocytogenes* to treatments when compared with *E. coli*. The inactivation rates for the sensitive cells (β) were higher when compared with the inactivation rates for the resistant cells (α) and ranged from 0.63 to 71.43 and 0.34 – 21.82, respectively for *E. coli* and *L. monocytogenes*. This implied a higher rate of inactivation during the first few minutes of treatment and more sensitive cells were weakened at shorter inactivation times, leaving a fraction of the resistant cells that were inactivated at a much lesser degree [27]. The data also demonstrated that *L. monocytogenes* were more resistant with low inactivation rates.

Several reactions can occur simultaneously that may lead to changes in the population of microorganisms during the inactivation process. According to Li et al. [58], these reactions can be sequential or parallel and their contributions to the overall resistance of these pathogens may vary. The biphasic model accounts for the presence of mixed strains or species with different resistances, which may be influenced by variations in the density of the stationary phase in the broth during inoculum preparation and/or the inoculation process [58]. Besides, interfering with organic matter in the fish can increase the resistance of bacteria as some cells may be located in a more hydrophobic and fatty environment. This was confirmed in a study suspending *B. cereus* spores in PFW containing bovine serum albumin (BSA) as a model organic matter, and these spores were more resistant to decontamination when compared with spores suspended in PFW without BSA [30]. Subpopulation with different resistances that were successfully predicted with the biphasic model were also reported for *E. coli*, *L. innocua*, *Saccharomyces cerevisiae* (*S. cerevisiae*), *Leuconostoc mesenteroides* (*L. mesenteroides*) and biofilms caused by *S. aureus* during US and ozone treatment [4], [27], [28], [54], [59].

#### 3.4.4 Log-logistic model

The kinetic parameters of the log-logistic model are also presented in Table 2. The log-logistic model is based on the presumption that microorganisms are composed of treatment-resistant spores, self-repair spores, and sensitive spores generated by the loss or absence of repair systems. The model proposes a treatment time parameter that is specific to bacteria strain and dependent on the treatment time so that the optimum inactivation time can be predicted [30]. The difference between the upper and lower asymptotes (*A*) and the maximum inactivation rate (*σ*) displayed a positive correlation with lethality and ranged from −0.91 to –33.34 and −0.08 to −13.79, respectively. It was suggested that the *A* value clearly lacks any biological or physical meaning [35], but the *σ* value further confirmed the improved inactivation from the combination treatments.

In contrast, the log time for achieving the maximum inactivation rate (*τ*) correlated inversely with lethality and UDWS presented the highest values at 4.63 and 4.85, while UPFBS presented the lowest values at 3.24 and 3.73 for *E. coli* and *L. monocytogenes*, respectively. According to Evelyn [37], the *τ* value typically decreases with increasing lethality and the lower values associated with *E. coli* in the current study suggested that the treatments were more lethal to *E. coli* when compared with *L. monocytogenes*. Similar trends were reported in the literature for the kinetic parameters of the log-logistic model. For example, values ranging from −1.908 to −4.252, −2.084 to −3.035 and 1.385 to 1.480 were reported for *A*, *σ*, and *τ* respectively, with increasing lethality when *B. cereus* spore suspension was added to PFW [30]. In another study, the values ranged from −2.47 to −3.4, −6.82 to −7.65, and 1.27 to 1.16 for *L. mesenteroides*, −1.97 to −2.53, −4.32 to −5.45, and 1.41 to 1.26 for *S. cerevisiae*, and −3.25 to −3.99, −3.71 to −4.67, and 1.43 to 1.27 for total coliforms, respectively for *A*, *σ*, and *τ*, with increasing ozone concentration and lethality during decontamination of sugarcane juice [27].

### 3.5 Selection of best fit model

The comparable better performances of Weibull, biphasic and log-logistic models for describing the inactivation kinetics of *E. coli* and *L. monocytogenes* in the current study necessitated additional statistical indicators for selecting the best fit model. Thus, the Akaike information criterion (AIC), Akaike increments (Δ_i_), and Akaike weights (W_i_), which are regarded as the best tools for easy ranking of models [27], were evaluated and results are presented in Table 3. The first-order kinetic model presented the smallest AIC for all treatment methods and the respective pathogens, and Δ_i_ = 0 for all cases signified substantial support for this model but received the lowest weightage values (W_i_) in the range of 4.99–5.24% and 5.12–5.39% for *E. coli* and *L. monocytogenes*, respectively. The Weibull model presented Δ_i_ values that were slightly >2 and ranged from 2.06 to 2.19 and 2.03–2.11 for *E. coli* and *L. monocytogenes*, respectively, and thus could also be categorized as having substantial support for consideration while making overall inference on the best fit model. The Weibull model presented fairly high weightage (W_i_) at values of 14.69–14.96% and 14.69–15.05% for *E. coli* and *L. monocytogenes*, respectively, and the values were higher when compared with the values for the first-order kinetic model. The biphasic and log-logistic models presented the highest Δ_i_ values, which were slightly higher than 4 with the minimum and maximum values of 3.97 and 4.19, respectively, showing relatively less support (4 ≤ Δ_i_ ≤ 7), but they presented the highest considerations for making inference with respect to the W_i_ values. Particularly, the biphasic model presented average values of 40.39 and 40.54%, and the log-logistic model presented average values of 39.68 and 39.33%, for *E. coli* and *L. monocytogenes*, respectively.

**Fig. 4:**
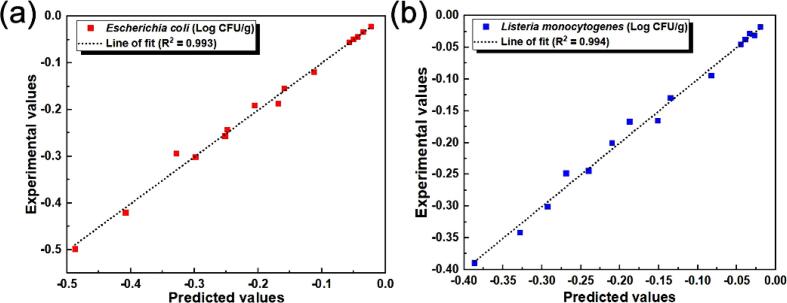
Correlation between experimental and predicted values according to the best fit biphasic model (a) *Escherichia coli* (b) *Listeria monocytogenes*.

Regarding the evidence ratio (E_R_), the models presented similar trends for both pathogens with the first-order kinetic model presenting the least likelihood of being selected over the other models at values of 0.13–0.35, closely followed by the Weibull model at values of 0.37–2.87. The biphasic model presented the most likelihood of being selected over the other models with values ranging from 1.02 to 7.83, closely followed by the log-logistic model at values of 0.97–7.69. Generally, the model with the minimum information criterion and increments, and highest weightage is considered as the best model for the particular data set [27], [41]. With respect to the AIC and Δ_i_, the models were ranked in the following order: first-order kinetics > Weibull > log-logistic > biphasic. In terms of the W_i_, and E_R_, they were ranked in the following order: biphasic > log-logistic > Weibull > first-order kinetics. Therefore, based on the relative support for each candidate model, weights of evidence, evidence ratio, and the overall performance in terms of adj-R^2^, the biphasic model was recognized as the best fit model for predicting the inactivation of both pathogens. A comparison of the scatter plot of the experimental and predicted data for the biphasic model is shown in Fig. 4, confirming good correlations with R^2^ ≥ 0.99 for both pathogens, which indicated close relationships between the observed and predicted data and demonstrated the accuracy of the selected best fit model.

## 4 Conclusions

The current study explored the suitability of the linear and non-linear models for describing the inactivation dynamics of *E. coli* and *L. monocytogenes* on grass carp as treated with ultrasound (US), plasma functionalized buffer (PFB) and their combinations (UPFB). Results indicated the limited bactericidal effects of US alone but suggested that US could further enhance the antibacterial properties of PFB with improved reductions during combination treatments. UPFB presented maximum reductions of 3.92 and 3.70 log CFU/g for *E. coli* and *L. monocytogenes*, respectively, and the interactions between treatments displayed potential synergy in the range of 0.18–0.50 log CFU/g. Statistical measurements of adj-R^2^, RMSE, and SSE, and validation indices of accuracy (A_f_) and bias (B_f_) factors indicated satisfactory and comparable model performance for the non-linear models, which were better fits in comparison with the linear model. Among the non-linear models, the biphasic model was the most accurate and reliable, as it presented the most likelihood of being selected with evidence ratios (E_R_) of 1.02–7.83 and 1.03–7.71 and moderate Akaike increments (Δ_i_) in 40.39 and 40.54% of the inactivation curves for *E. coli* and *L. monocytogenes*, respectively. The findings should provide fundamental background for performance evaluation and optimization of the hygiene management systems during fish processing for guaranteeing safe supply.

### CRediT authorship contribution statement

**Okon Johnson Esua:** Writing – original draft, Formal analysis, Investigation. **Da-Wen Sun:** Supervision, Funding acquisition, Resources, Writing – review & editing. **Clement Kehinde Ajani:** Investigation. **Jun-Hu Cheng:** Validation, Funding acquisition, Resources. **Kevin M. Keener:** Investigation.

## Declaration of Competing Interest

The authors declare that they have no known competing financial interests or personal relationships that could have appeared to influence the work reported in this paper.
